# Dietary L‐Tryptophan Improves Growth, Antioxidant Status, Intestinal Barrier, and Resistance to *Aeromonas hydrophila* Infection in Largemouth Bass

**DOI:** 10.1155/anu/8131057

**Published:** 2026-10-25

**Authors:** Xindang Zhang, Hengzhi Wang, Chongchong Guo, Shikun Feng, Xue Tian, Yanmin Zhang, Xulu Chang, Guokun Yang, Mohammed A. E. Naiel, Xiaolin Meng

**Affiliations:** ^1^ College of Fisheries Henan Normal University Xinxiang China henannu.edu.cn; ^2^ Engineering Technology Research Center of Henan Province for Aquatic Animal Cultivation Henan Normal University Xinxiang China henannu.edu.cn; ^3^ Tongwei Agricultural Development Co. Ltd. Chengdu China; ^4^ Animal Production Department Zagazig University Zagazig Egypt zu.edu.eg

**Keywords:** antioxidant status, intestinal barrier, largemouth bass, tryptophan

## Abstract

Tryptophan (Trp) has been well documented to positively regulate nutrient metabolism, immune responses, and intestinal microecological homeostasis in mammals. However, relevant studies regarding its functions in largemouth bass remain limited. Herein, we assessed the impacts of dietary Trp on growth, antioxidant status, intestinal barrier, and disease resistance in largemouth bass. Fish were fed a basal control diet (C) or basal diets supplemented with 0.1%, 0.2%, 0.4%, and 0.8% L‑Trp for 60 days. Dietary L‐Trp supplementation significantly improved FBW, WGR, and SGR of largemouth bass, with the maximum values found in the 0.4%Trp group. Based on the WGR and SGR data, quadratic regression analysis further identified 0.49% as the optimal dietary L‐Trp inclusion level. In terms of antioxidant capacity, the 0.4%Trp group exhibited markedly higher serum and intestinal activities of CAT, GSH‐PX, and SOD, as well as a higher content of T‐AOC, compared to the C group. Moreover, the 0.4%Trp group significantly upregulated intestinal *Nrf2* expression and downregulated *Keap1* expression. Relative to the C group, dietary Trp supplementation significantly elevated intestinal villus height and muscular layer thickness in the 0.1%, 0.2%, and 0.4% Trp groups. Consistently, the mRNA levels of intestinal tight junction genes (*ZO-1*, *Claudin-1*, and *Claudin-4*) were upregulated in the 0.4%Trp group. Supplementation with 0.4%Trp significantly downregulated the expression of proinflammatory cytokines (*TNF-α*, *IL-1β*, and *IL-8*) and upregulated the expression of antiinflammatory cytokines (*TGF-β1*, *IL-10*, *IL-22*, and *AHR*). Intestinal microbial analysis further revealed that dietary L‐Trp supplementation enriched the abundance of beneficial bacteria, reduced pathogenic microbial populations, and improved intestinal microbial diversity and richness. Finally, dietary L‐Trp supplementation effectively improved the survival rate of largemouth bass following an *Aeromonas hydrophila* challenge. This was associated with enhanced serum activities of LZM, AKP, and ACP, as well as increased intestinal T‐SOD activity. Additionally, L‐Trp supplementation upregulated the expression of *Nrf2*, *TGF-β1*, *IL-10*, and *IL-22*. In contrast, the 0.4%Trp group reduced the MDA content and downregulated expression of *Keap1*, *HSP70*, *TNF-α*, *IL-1β*, and *IL-8*. Collectively, 0.4% dietary Trp strengthens antioxidant defense, remodels intestinal microbiota, improves intestinal physical and immune barriers, and enhances resistance against *A. hydrophila* infection in largemouth bass.

**Summary**

• Dietary 0.4% tryptophan improved the growth performance and preserved intestinal barrier function and health.
• Tryptophan supplementation enhances systemic antioxidant capacity and resistance to *Aeromonas hydrophila* infection.
• Dietary tryptophan represents a promising functional feed additive for healthy largemouth bass aquaculture.

## 1. Introduction

Largemouth bass (*Micropterus salmoides*) is a commercially vital carnivorous freshwater fish in China, widely cultivated for its fast growth rate, superior flesh quality, and considerable economic benefits [1, 2]. In recent years, the booming development of intensive and high‐density aquaculture systems has led to a series of challenges, including compromised growth performance, reduced physiological fitness, and increased susceptibility to diseases [3, 4]. Nutritional management practices for largemouth bass have often prioritized maximum yield, frequently resulting in overfeeding with high‐protein and high‐fat diets [5, 6]. This approach imposes an excessive metabolic burden on the intestine, thereby damaging its structural integrity and physiological function [7–9]. The consequent development of digestive disorders and a systemic subhealth status not only compromises fish health but also severely impedes the sustainable advancement of the aquaculture sector [10]. Therefore, exploring safe and efficient nutritional regulation strategies to improve the growth, intestinal status, and disease tolerance of largemouth bass has become a research hotspot in aquatic nutrition.

Amino acid nutrition is central to the growth and physiological metabolism of fish [11]. As essential nutrients, functional amino acids act not merely as precursors for protein synthesis; they also mediate antioxidant capacity, intestinal barrier function, and immune responses [12–14]. Tryptophan (Trp), an essential amino acid for fish growth, cannot be endogenously synthesized and must be obtained exclusively from feed and dietary nutrients [15, 16]. In addition, tryptophan is also critical for fish metabolism, development, and stress regulation. As reported by NRC (2011) [17], the dietary tryptophan requirement for optimal fish growth ranges from 3.0 to 12.0 g/kg. For instance, the optimal dietary Trp level for rainbow trout (*Oncorhynchus mykiss*) is 4.0 g/kg diet [18], Japanese flounder (*Paralichthys olivaceus*) is 5.0 g/kg diet [19], hybrid grouper (*Epinephelus fuscoguttatus ♀* × *E. lanceolatus*♂) is 4.1–4.6 g/kg diet [20], and silver catfish (*Rhamdia quelen*) is 2.5–3.4 g/kg diet [21]. Furthermore, earlier investigations have revealed that Trp not only acts as a fundamental substrate for protein anabolism but also generates a wide range of bioactive substances to modulate neural signal transduction, endocrine regulation, immune reactions, and redox balance [22–24]. Moreover, Trp also exerts beneficial effects on improving intestinal barrier integrity and maintaining intestinal health [25–28]. Intestinal health is a critical determinant of growth performance and disease resistance of fish, and it is closely associated with intestinal morphology, antioxidant enzyme activities, tight junction gene expression, and intestinal microbiota composition [29, 30]. Moreover, earlier findings have revealed that dietary Trp supplementation improves disease resistance and decreases mortality in aquaculture species subjected to intensive rearing [31, 32]. These results emphasize the importance of dietary Trp as a vital nutritional component for optimizing fish health and growth performance in high‐density farming systems.

Herein, we investigated the impacts of graded dietary tryptophan levels on growth, antioxidant status, and intestinal barrier integrity in largemouth bass. Pathogen challenge assays were performed to verify the protective role of Trp during *Aeromonas hydrophila* infection. Our objectives were to reveal the underlying mechanisms by which dietary Trp improves growth, sustains intestinal homeostasis, and strengthens disease resistance and to identify the optimal supplementation dose. The obtained data will lay a theoretical foundation for utilizing Trp as an eco‐friendly feed additive in sustainable largemouth bass aquaculture and shed new light on nutritional immune regulation and disease prevention in carnivorous freshwater fish.

## 2. Materials and Methods

### 2.1. Experimental Diets and Animal Feeding

The ingredient formulation and proximate composition of the five experimental diets are summarized in Table 1. Fish meal, chicken meal, pork meal, and soybean meal were adopted as the main protein ingredients, with dietary lipid supplied by fish oil, soybean oil, and soybean lecithin. Five dietary treatments were established in total: a basal control diet (Group C) and four test diets formulated by supplementing the basal recipe with graded supplementation levels of L‐tryptophan (0.1%, 0.2%, 0.4%, and 0.8%), corresponding to the 0.1%Trp, 0.2%Trp, 0.4%Trp, and 0.8%Trp groups, respectively. The fish meal, chicken meal, pork meal, soybean meal, and bran in the feed raw materials were ground into powder by a 30B high‐efficiency universal grinder, and then the feed raw materials were manually mixed evenly according to the feed formula in Table 1. The mash was pelleted using a laboratory granulator to produce feed pellets with a diameter of ~4.0 mm. The feed pellets were air‐dried in a dry, ventilated area, subsequently sealed in plastic bags, and stored at −20°C until use.

**Table 1:** Formulations of the experimental diets (% dry matter).

Ingredients	Group				
	C	0.1%Trp	0.2%Trp	0.4%Trp	0.8%Trp
Fish meal^a^	30.00	30.00	30.00	30.00	30.00
Chicken meal^a^	6.00	6.00	6.00	6.00	6.00
Pork meal^a^	6.00	6.00	6.00	6.00	6.00
Soybean meal^a^	24.00	24.00	24.00	24.00	24.00
Wheat bran	12.35	12.25	12.15	11.95	11.55
Wheat flour^a^	14.00	14.00	14.00	14.00	14.00
L‐tryptophan	0.00	0.10	0.20	0.40	0.80
Fish oil	1.50	1.50	1.50	1.50	1.50
Soybean oil	0.50	0.50	0.50	0.50	0.50
Soyabean lecithin	1.50	1.50	1.50	1.50	1.50
Ca(H_2_PO_4_)_2_	1.50	1.50	1.50	1.50	1.50
Choline chloride	0.50	0.50	0.50	0.50	0.50
Ethoxyquin	0.05	0.05	0.05	0.05	0.05
Vitamin C	0.10	0.10	0.10	0.10	0.10
Mineral premix^b^	1.00	1.00	1.00	1.00	1.00
Vitamin premix^c^	1.00	1.00	1.00	1.00	1.00
Total	100.00	100.00	100.00	100.00	100.00
Proximate composition					
Tryptophan	0.41	0.46	0.55	0.74	1.11
Crude lipid	11.08	11.09	11.13	11.20	11.34
Crude protein	45.33	45.39	45.64	45.84	46.25
Ash	12.17	13.22	12.57	11.20	12.05
Moisture	6.13	6.20	6.53	6.73	6.57

^a^Fish meal, crude protein, 66.83%, crude lipid, 10.68%; chicken meal, crude protein, 65.02%, crude lipid, 11.68%; pork meal, crude protein, 71.65%, crude lipid, 13.52%; soybean meal, crude protein, 48.98%, crude lipid, 2.11%; wheat flour, crude protein, 15.95%, crude lipid, 4.26%.

^b^Mineral premix (mg/kg diet): FeSO_4_∙H_2_O 4800 mg, CuSO_4_ 3120 mg, ZnSO_4_ 1440 mg, MnSO_4_ 3720 mg, Ca (IO_3_)_2_ 100 mg, KI 200 mg, Na_2_SeO_3_ 84 mg, COCl_2_ 48 mg, and MgSO_4_∙H_2_O 500 mg.

^c^Vitamin premix (mg/kg diet): VA 532,000 IU, VD_3_ 130,000 IU, VE 7000 mg, VB_1_ 1500 mg, VB_2_ 1800 mg, VB_6_ 65 mg, niacinamide 230 mg, calcium‐pantothenate 350 mg, and inositol 1000 mg.

Prior to the initiation of the 60‐day feeding experiment, all largemouth bass were acclimatized in the intelligent greenhouse recirculating aquaculture system at the College of Fisheries, Henan Normal University. Following acclimation, a total of 450 healthy largemouth bass (mean initial body weight, 16.19 ± 0.06 g) were randomly distributed to five dietary treatments, each comprising three replicate tanks (30 fish per tank). Two daily feeding sessions were conducted at 08:00 and 17:00. The daily feed ration, feed intake, and growth performance were recorded for each tank. Over the experimental period, pH remained within 7.5 to 7.6, dissolved oxygen concentration remained above 8.0 mg/L, and approximately one‐third of the total water volume was exchanged on a daily basis.

### 2.2. Sample Collection

All test fish underwent 24 h of feed deprivation following the 60‐day feeding trial. All individuals were harvested by netting, counted, and weighed for the calculation of growth parameters. Anesthesia with tricaine methane sulfonate (MS‐222) was performed on all fish prior to tissue collection. Following anesthesia, the body weight and length were measured for nine fish prior to dissection on an ice‐chilled tray for the computation of morphological indices.

Blood sampling was performed on nine randomly chosen fish from each tank, with the obtained samples being transferred into sterile centrifuge tubes. Midgut and foregut samples were aseptically excised from nine fish per tank and immediately placed in sterile nuclease‐free tubes. The midgut samples were designated for analyzing intestinal immunity and tight junction protein gene expression, whereas the foregut samples were allocated for digestive enzyme activity analysis. For histological examination, midgut tissues were collected from three fish per tank. Additionally, intestinal contents were sampled from six other randomly selected fish per tank for bacterial DNA extraction to be subsequently used for analyzing gut microbiota diversity.

### 2.3. Chemical Analysis

#### 2.3.1. Composition Analysis

Both the experimental diets and whole‐body samples were oven‐dried at 105°C to a constant weight for moisture content determination. The crude lipid content was quantified using the standard ether extraction method. The Kjeldahl method was employed to determine the crude protein content using a nitrogen‐to‐protein conversion factor of 6.25. The ash content was quantified via high‐temperature ashing in a muffle furnace held at 550°C. The dietary tryptophan content was determined by high‑performance liquid chromatography (HPLC) according to GB/T 15400‑2018. Briefly, 50–100 mg of the sample (weighed accurately to 0.0001 g) was transferred into an anaerobic bottle. Then, 1.5 mL of lithium hydroxide solution was added, followed by nitrogen flushing for 1 min. The bottle was tightly sealed and placed in an oven for hydrolysis at 110 ± 2°C for 20 h. After hydrolysis, the anaerobic bottle was taken out and cooled to room temperature. The hydrolysate was quantitatively transferred into a 25 mL brown volumetric flask using a sodium acetate buffer solution, and the solution was brought to volume with the same buffer. The solution was centrifuged or filtered through a 0.45 μm membrane filter prior to HPLC analysis.

#### 2.3.2. Biochemical Analysis

Intestinal tissues were accurately weighed on an electronic balance (precision: 0.1 g) and homogenized in nine volumes of precooled 0.9% saline to yield 10% (w/v) homogenates with a high‐throughput tissue homogenizer. Following centrifugation at 4000 rpm for 15 min, the resultant supernatants were collected for biochemical determinations. The activities of trypsin (Number A080‐2‐2), lipase (Number A054‐1‐1), and α‐amylase (Number C016‐1‐1), as well as the concentrations of malondialdehyde (MDA, Number A003‐1‐2), catalase (CAT, Number A007‐1‐1), superoxide dismutase (SOD, Number A001‐2‐2), glutathione peroxidase (GSH‐Px, Number A005‐1‐2), and total antioxidant capacity (T‐AOC, Number A015‐1‐2) were quantified using commercial assay kits from Nanjing Jiancheng Bioengineering Institute (Nanjing, China). All analytical procedures were performed in strict compliance with the manufacturer’s standard operating protocols. Soluble protein concentrations in the supernatants were determined via the Bradford method to normalize all enzyme activity values.

#### 2.3.3. Histological Analysis

Fixed midgut samples were processed following routine histological protocols. After serial dehydration in graded ethanol solutions, the tissues were cleared with xylene and embedded in paraffin. H&E staining was performed on processed tissue sections for histomorphological assessment. Intestinal cross‑sections were examined with a Zeiss microscope (Germany) for quantification of villus height and muscular layer thickness. Villus height represented the vertical distance from the villus tip to the crypt opening, and muscular‑layer thickness was measured in representative tissue regions.

#### 2.3.4. Quantitative Real‐Time PCR Analysis

The TRIzol reagent purchased from Servicebio (Wuhan, China) was employed for total RNA extraction from midgut tissue samples. The resulting RNA pellets were dissolved in RNase‐free water, and their concentration and integrity were verified via Nano Drop 2000 spectrophotometry and agarose gel electrophoresis, respectively. A commercial reverse transcription kit manufactured by Accurate Biology (Hunan, China) was utilized to convert qualified RNA samples into complementary DNA (cDNA), with all procedures strictly adhering to the manufacturer’s standardized protocols. For RT‐qPCR analysis, all reactions were subsequently prepared with SYBR Green Supermix and run on a LightCycler 480 real‐time PCR system. The 2^−ΔΔCT^ method was applied to determine the relative mRNA expression levels of target genes, with 18S rRNA serving as the endogenous reference gene for data normalization. Full details of all primer sequences are presented in Table 2.

**Table 2:** Primer sequences used for quantitative real‑time PCR analysis.

Gene	Accession number	Forward (5′→3′)	Reverse (5′→3′)
*18S*	XM_038704162.1	GTTCTATTTTGTGGGTTT	CCTCCGACTTTCGTTCTT
*Hsp70*	XM_038710012.1	GTCCTACGCCTTCAACACGA	GCTGATGGTCTCGTCACACT
*Nrf2*	XM_038720536.1	TCACCAAAGACAAGCGTAA	CAGGCAGATTGATAATCATAGA
*Keap1*	XM_038728592.1	GCACCTAACCGTGGAACTCAA	CCAGTTTTAGCCAGTCATTGTTCC
*TNF-α*	XM_038710731.1	CTTCGTCTACAGCCAGGCATCG	TTTGGCACACCGACCTCACC
*IL-1β*	XM_038733429.1	GTAGTCCCGTTGTTCT	ATTGCTCCAGTTTCAG
*IL-8*	XM_038713528.1	CTCTATCGTGGTGCTCC	TGGGTTTGCTCTCTGTC
*TGF-β1*	XM_038693206.1	AGGTGATGCCGATGGATGGATAG	TGGAATGACAACAGTAGCAGGAGT
*IL-10*	XM_038696252.1	CGGCACAGAAATCCCAGAGC	CAGCAGGCTCACAAAATAAACATCT
*IL-22*	XM_038709272.1	CCTCCCTGGAGAAGAGCTAC	AGGTAAGTGAACAGGATGTCGA
*ZO-1*	XM_038701018.1	ATCTCAGCAGGGATTCGACG	CTTTTGCGGTGGCGTTGG
*AHR*	XM_038715445.1	AACTGAAGGTGCCAAGTCCAA	GGAGAACAGGTGATGGTTGAGG
*CYP1A1*	XM_038701222.1	CCAAAGCCCCTGCCTATCAT	TGACTGTGACCCATACGGTG
*Claudin-1*	XM_038713307.1	CCAGGGAAGGGGAGCAATG	GCTCTTTGAACCAGTGCGAC
*Claudin-4*	XM_038708626.1	TAATCGCTATGGTGGGAGCC	GCCCCGATCTCCATCTTCTG
*Caspase-3*	XM_038713063.1	GCTTCATTCGTCTGTGTTC	CGAAAAAGTGATGTGAGGTA
*Bax*	XM_038704178.1	TCTTCACTCAGTCCCACAAA	ATACCCTCCCAGCCACC
*Bcl-2*	XM_038723143.1	CCATCCACGACGAACCTG	GGCGTATCGCTGCTCAAACT
*IDO1*	XM_038704041.1	ATGATACACACGGTGCTCCAA	CGCAGGGCAATATCCATCCA
*NMNAT1*	XM_038722812.1	TGACATCTCAGCCACTCACG	CTCTAGAACCACGTCGGCAT
*QPRT*	XM_038714452.1	ATCGGTCCAGATGCCATCAC	CATTGCCTGCAGCTGAGAAC
*mTOR*	XM_038723321.1	GCGTATCGTAGAGGACTGGC	GTCAACACCGAGAAGGAGCA
*S6K1*	XM_038708508.1	TCGTTCGAGCCAAAGGTCC	TCGATACCTCCATGGGTTGC
*4EBP1*	XM_038703877.1	ACGAGGTCTGCCCAACATTC	CAGCGTTGCTGCTATCAGGT

#### 2.3.5. Intestinal Contents 16S rDNA Analysis

Intestinal content samples from the midgut and hindgut were collected 12 h after the final feeding (*n* = 6). Bacterial DNA was isolated from intestinal mucosa specimens with the QIAamp DNA stool mini kit. DNA concentration and purity were evaluated via a NanoDrop 2000 spectrophotometer (Thermo, USA), while DNA integrity was verified using 1 % agarose gel electrophoresis. Samples meeting quality criteria were submitted to Nanjing Personal Biotechnology Co., Ltd., for 16S rRNA gene sequencing. The V3–V4 hypervariable region of the 16S rRNA gene was amplified with the specific primer pair 341F (5′‑CCTACGGGNGGCWGCAG‑3′) and 806R (5′‑GGACTACHVGGGTATCTAAT‑3′). Amplicons from the second‑round PCR were purified with AMPure XP Beads, quantified on an ABI StepOnePlus Real‑Time PCR system (Life Technologies, USA), and finally sequenced on a NovaSeq 6000 platform under the PE250 pooled‑sequencing mode.

Following sequencing, raw reads were processed by filtering low‑quality sequences and assembling paired‑end reads into tags. The resulting clean tags were clustered, and chimeric sequences identified during clustering comparisons were eliminated. After OTU generation, high‑throughput bioinformatic analyses were conducted on the Gidio Bioplatform.

### 2.4. *Aeromonas Hydrophila* Challenge Trial

Following the feeding trial, a 7‐day *Aeromonas hydrophila* (*A. hydrophila*) challenge trial was performed. The *A. hydrophila* strain used in this study was preserved in the Laboratory of Aquatic Microbial Ecology and Resource Utilization, College of Fisheries, Henan Normal University. The strain was cultured in LB medium at 28°C for 12 h. Following bacterial cell collection by centrifugation at 3000 ×*g* for 5 min, the resulting pellet was resuspended in sterile PBS to generate a working bacterial suspension for the subsequent bacterial challenge. Prior to the formal challenge experiment, the median lethal dose (LD_50_) of *A. hydrophila* for juvenile largemouth bass was determined following the method described by Yun et al. [33]. The formal challenge experiment included negative control (NC), positive control (C), and 0.1 %, 0.2 %, 0.4 %, and 0.8 % Trp‑supplemented groups. Fifteen largemouth bass were used per group. Each fish in the NC group was intraperitoneally injected with 200 μL PBS, while the remaining groups received 200 μL of *A. hydrophila* suspension (1 × 10^7^ CFU/mL) via an intraperitoneal injection. During the 7‐day challenge period, feeding and rearing management were consistent with those in the previous feeding trial (Section 2.1). Fish mortality was monitored at 12‐h intervals; dead fish were promptly removed and checked for typical symptoms of *A. hydrophila* infection. The cumulative fish survival rate was calculated according to the mortality records throughout the challenge period.

### 2.5. Calculation of Growth Performance Parameters

Weight gain rate (WGR, %)=final weight−initial weightinitial weight×100Specific growth rate (SGR, %)=Lnfinal weightinitial weightdays×100Feed conversion rate (FCR, %)=total feed consumptionfinal weight−initial weight×100Condition factor (K, g/cm3)=final weightlength of fish3×100.

### 2.6. Statistical Analysis

Statistical analyses were performed using SPSS 26.0 (IBM, USA). The normality and homogeneity of variances were first verified. One‐way analysis of variance (ANOVA) was carried out, and multiple comparisons were conducted using Tukey’s HSD posthoc test. All results are presented as mean ± SEM deviation, with statistical significance defined at *p*  < 0.05.

## 3. Results

### 3.1. Growth Performance and Whole‐Body Composition

Dietary Trp supplementation significantly elevated FBW, WGR, and SGR relative to the C group, with the maximum values exhibited in the 0.4%Trp treatment (*p* < 0.05, Table 3). In contrast, FCR was markedly reduced in 0.4%Trp treatment relative to 0.1% and 0.2%Trp treatments (*p* < 0.05). The K was significantly increased in fish fed the 0.2%, 0.4%, and 0.8%Trp diets relative to the C group (*p* < 0.05). No statistically significant differences in survival rate were detected across all treatments (*p* > 0.05). As presented in Figure 1, quadratic polynomial regression was performed based on WGR (Figure 1a) and SGR (Figure 1b) data, and the optimal dietary Trp‐supplementation level for largemouth bass was estimated to be 0.49% in the present trial.

**Figure 1:**
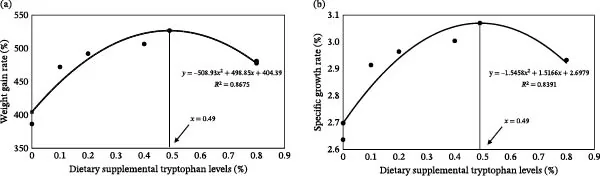
Regression analysis between dietary tryptophan level and weight gain rate (a) and specific growth rate (b) of largemouth bass.

**Table 3:** Effects of L‐tryptophan on growth performance of largemouth bass.

Growth index	Group				
	C	0.1%Trp	0.2%Trp	0.4%Trp	0.8%Trp
IBW (g)	16.15 ± 0.01	16.24 ± 0.02	16.21 ± 0.03	16.18 ± 0.05	16.15 ± 0.05
FBW (g)	77.63 ± 1.79^a^	94.03 ± 3.72^b^	95.48 ± 3.48^b^	97.80 ± 2.54^c^	93.40 ± 3.36^b^
WGR (%)	380.54 ± 2.96^a^	479.04 ± 5.87^b^	489.10 ± 1.55^bc^	504.52 ± 8.89^c^	478.41 ± 5.09^b^
SGR (%/d)	2.62 ± 0.01^a^	2.93 ± 0.02^b^	2.95 ± 0.01^bc^	3.00 ± 0.03^c^	2.93 ± 0.01^b^
FCR	1.14 ± 0.01^ab^	1.15 ± 0.02^b^	1.16 ± 0.02^b^	1.12 ± 0.02^a^	1.14 ± 0.02^ab^
K (g/cm^3^)	2.35 ± 0.04^a^	2.44 ± 0.04^ab^	2.54 ± 0.04^b^	2.50 ± 0.04^b^	2.55 ± 0.05^b^
Survival (%)	100.00 ± 0.00	100.00 ± 0.00	100.00 ± 0.00	100.00 ± 0.00	100.00 ± 0.00

*Note*: Data are presented as mean ± SEM (*n* = 9). Values within the same row bearing dissimilar superscript letters differ significantly at *p*  < 0.05.

As shown in Table 4, whole‐body crude protein was markedly higher in all Trp‐supplemental groups relative to the C group, with the 0.4%Trp treatment presenting the maximum value and exceeding all other supplemental treatments (*p* < 0.05). Whole‐body crude lipid levels were markedly reduced in the 0.1%, 0.2%, and 0.4%Trp treatments relative to group C (*p* < 0.05). Whole‐body ash and moisture levels exhibited no significant variations across dietary Trp treatments (*p* > 0.05).

**Table 4:** Effects of L‐tryptophan on whole‐body composition of largemouth bass.

Whole‐body composition	Group				
	C	0.1%Trp	0.2%Trp	0.4%Trp	0.8%Trp
Crude protein (% DM)	53.97 ± 0.28^a^	55.42 ± 0.15^b^	56.59 ± 0.48^c^	58.41 ± 0.21^d^	56.73 ± 0.21^c^
Crude lipid (% DM)	27.43 ± 0.31^b^	26.50 ± 0.05^a^	26.50 ± 0.10^a^	26.27 ± 0.10^a^	26.84 ± 0.09^ab^
Ash (% DM)	16.90 ± 0.09	16.83 ± 0.05	16.93 ± 0.06	16.92 ± 0.05	16.90 ± 0.02
Moisture	66.78 ± 0.44	67.66 ± 0.18	67.92 ± 0.36	66.76 ± 0.12	67.34 ± 0.79

*Note*: Data are presented as mean ± SEM (*n* = 6). Values within the same row bearing dissimilar superscript letters differ significantly at *p*  < 0.05.

### 3.2. Antioxidant Status

The serum and intestinal antioxidant indices are revealed in Table 5. Relative to the C group, serum MDA contents were significantly decreased in all Trp‐supplemented groups, with the most prominent reduction detected in 0.4%Trp treatment (*p* < 0.05). Conversely, serum CAT, SOD, and GSH‐Px activities, together with T‐AOC levels, were significantly elevated in all Trp treatments relative to group C, with the 0.4% Trp treatment showing the most pronounced increase (*p* < 0.05).

**Table 5:** Effects of L‐tryptophan on serum and intestinal antioxidant capacity of largemouth bass.

Index	Group				
	C	0.1%Trp	0.2%Trp	0.4%Trp	0.8%Trp
*Serum*					
MDA (nmol/mL)	17.51 ± 0.85^c^	13.83 ± 0.62^b^	13.73 ± 0.47^b^	9.39 ± 0.74^a^	13.87 ± 0.73^b^
CAT (U/mL)	13.42 ± 1.50^a^	19.52 ± 1.44^b^	21.25 ± 1.28^b^	26.53 ± 0.89^c^	18.21 ± 1.01^b^
SOD (U/mL)	46.87 ± 2.35^a^	53.29 ± 1.34^b^	55.39 ± 1.83^b^	61.16 ± 1.92^c^	52.65 ± 1.91^b^
T‐AOC (U/mL)	6.74 ± 0.48^a^	8.56 ± 0.71^b^	8.71 ± 0.28^b^	11.03 ± 0.70^c^	10.74 ± 0.40^c^
GSH‐PX (U/mL)	176.96 ± 5.17^a^	194.53 ± 4.03^b^	196.92 ± 7.70^b^	251.78 ± 5.90^c^	209.17 ± 5.92^b^
*Intestinal*					
MDA (nmol/mg prot)	2.61 ± 0.27	2.11 ± 0.13	2.04 ± 0.09	2.11 ± 0.11	2.36 ± 0.22
CAT (U/mg prot)	8.24 ± 0.49^a^	9.97 ± 0.47^b^	10.00 ± 0.82^b^	12.92 ± 0.34^c^	9.65 ± 0.68^b^
SOD (U/mg prot)	37.96 ± 1.67^a^	42.12 ± 1.24^b^	44.65 ± 0.96^b^	50.38 ± 1.15^c^	42.01 ± 1.43^b^
T‐AOC (U/mg prot)	1.63 ± 0.09^a^	3.91 ± 0.50^b^	3.94 ± 0.47^b^	4.74 ± 0.50^b^	3.89 ± 0.61^b^
GSH‐PX (U/mg prot)	130.10 ± 8.21^a^	153.83 ± 8.93^a^	162.62 ± 10.38^ab^	201.78 ± 24.07^b^	160.36 ± 16.65^ab^

*Note*: Data are presented as mean ± SEM (*n* = 9). Values within the same row bearing dissimilar superscript letters differ significantly at *p*  < 0.05.

For intestinal antioxidant status, the CAT and SOD activities and T‐AOC levels were substantially higher in all Trp treatments than in the C group, with the maximum increase detected in 0.4%Trp treatment (*p* < 0.05). Moreover, the GSH‐Px activity in 0.4%Trp treatment was markedly higher than that in C and 0.1%Trp treatments (*p* < 0.05). No significant difference in intestinal MDA content was exhibited across all experimental treatments (*p* > 0.05).

The expression of *Nrf2* was markedly upregulated in fish fed 0.2% and 0.4%Trp diets relative to the C group (*p* < 0.05, Figure 2). Conversely, transcript abundances of *Keap1* and *HSP70* were markedly downregulated in all Trp‐supplemented groups relative to the group C (*p* < 0.05).

**Figure 2:**
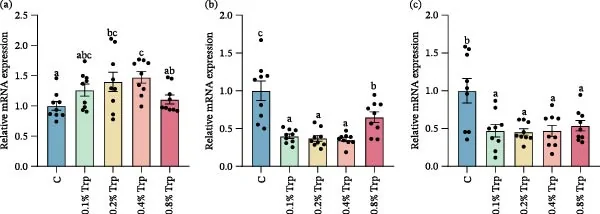
Effects of L‐tryptophan on the expression of intestinal antioxidant genes in largemouth bass. Data are presented as mean ± SEM (*n* = 9). Values within the same row bearing dissimilar superscript letters differ significantly at *p*  < 0.05. (a) *Nrf2*, (b) *Keap1*, and (c) *Hsp70*.

### 3.3. Intestinal Digestive Enzyme Activities

Table 6 summarizes the digestive enzyme activities of largemouth bass. Fish fed the 0.4%Trp diet exhibited significantly higher lipase and trypsin activities relative to the C group (*p* < 0.05). Furthermore, α‐amylase activity was notably increased in the 0.1%, 0.2%, and 0.4%Trp treatments compared with group C and 0.8%Trp group (*p* < 0.05).

**Table 6:** Effects of L‐tryptophan on intestinal digestive enzyme activity of largemouth bass.

Index	Group				
	C	0.1%Trp	0.2%Trp	0.4%Trp	0.8%Trp
Lipase (U/mg prot)	5.97 ± 0.42^a^	6.42 ± 0.78^ab^	6.41 ± 0.44^ab^	7.86 ± 0.55^b^	6.15 ± 0.24^a^
Trypsin (U/g prot)	2.66 ± 0.13^a^	3.26 ± 0.21^ab^	3.32 ± 0.28^ab^	3.63 ± 0.25^b^	3.13 ± 0.24^ab^
α‐Amylase (U/mg prot)	0.22 ± 0.01^a^	0.25 ± 0.01^b^	0.24 ± 0.01^b^	0.24 ± 0.01^b^	0.23 ± 0.01^a^

*Note*: Data are presented as mean ± SEM (*n* = 9). Values within the same row bearing dissimilar superscript letters differ significantly at *p*  < 0.05.

### 3.4. Intestinal Physical Barrier

Compared with group C, all Trp‐supplemented groups showed intact and tightly arranged intestinal villi (Figure 3a). Villus height and muscular layer thickness were notably elevated in the 0.1%, 0.2%, and 0.4%Trp treatments compared to those in group C (*p* < 0.05, Figure 3b,c). Similarly, the expressions of *ZO-1* and *Claudin‑1* were significantly upregulated in the 0.1%, 0.2%, and 0.4%Trp treatments relative to group C (*p* < 0.05, Figure 3d,e). Furthermore, the expression level of *Claudin‑4* was notably increased in all Trp‐supplemented treatments relative to the C group (*p* < 0.05, Figure 3f).

**Figure 3:**
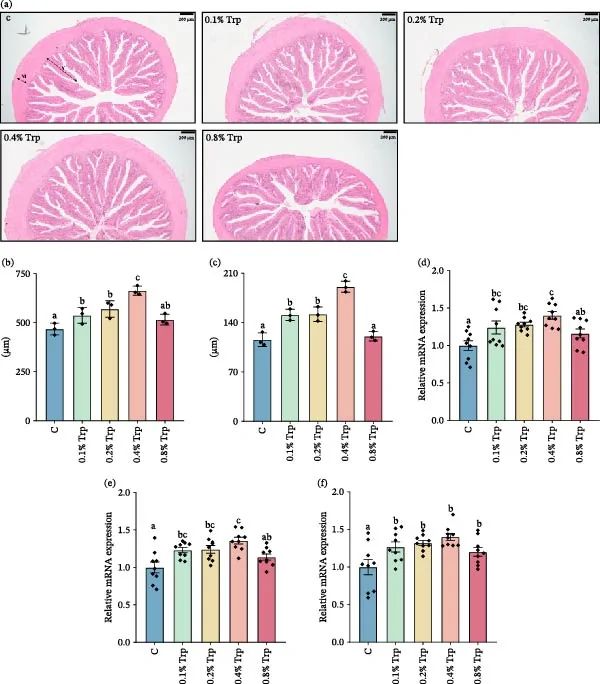
Effects of L‐tryptophan on intestinal tissue morphology of largemouth bass. (a) Midgut H&E staining ×50 (*n* = 3), Bar = 200 μm. V: Villus, M: Muscularis. (b) Villus height, (c) muscular thickness, (d) *ZO-1*, (e) *Claudin-1*, and (f) *Claudin-4*. Data are presented as mean ± SEM (*n* = 9). Values within the same row bearing dissimilar superscript letters differ significantly at *p*  < 0.05.

### 3.5. Intestinal Immunological Barrier

Dietary Trp supplementation significantly suppressed the expression of *TNF-α*, *IL-1β*, and *IL-8* relative to the C group, with the strongest downregulation detected in the 0.4%Trp treatment (*p* < 0.05, Figure 4a–c).

**Figure 4:**
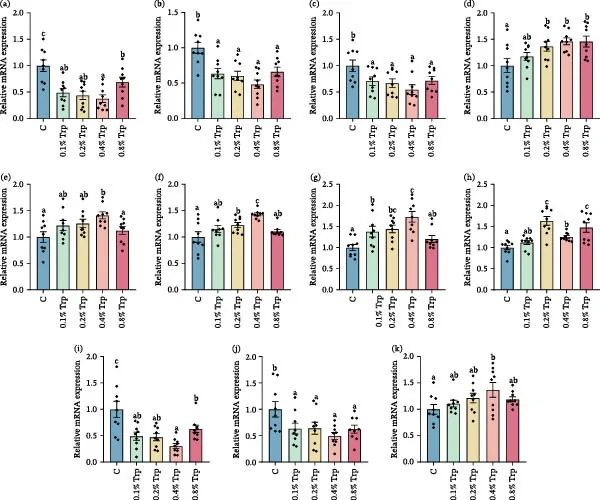
Effects of L‐tryptophan on the expression of intestinal immune‐related genes in largemouth bass. (a) *TNF-α*, (b) *IL-1β*, (c) *IL-8*, (d) *AHR*, (e) *TGF-β1*, (f) *IL-10*, (g) *IL-22*, (h) *CYP1A1*, (i) *Caspase-3*, (j) *Bax*, and (k) *Bcl-2*. Data are presented as mean ± SEM (*n* = 9). Values within the same row bearing dissimilar superscript letters differ significantly at *p*  < 0.05.

Significantly higher transcript levels of *TGF-β1* were observed in fish receiving the 0.4%Trp diet relative to the C and 0.8%Trp treatments (*p* < 0.05, Figure 4e). Similarly, transcript levels of *IL-10* and *IL-22* were markedly elevated in the 0.4%Trp treatment compared with all other dietary treatments (*p* < 0.05, Figure 4f,g). Furthermore, dietary Trp inclusion at 0.2%, 0.4%, and 0.8% significantly upregulated the transcript levels of *AHR* and *CYP1A1* relative to the C group (*p* < 0.05, Figure 4d,h).

Dietary Trp supplementation significantly downregulated the transcript levels of proapoptotic genes *Caspase-3* and *Bax* in all treatments compared with the C group (*p* < 0.05, Figure 4i,j). Conversely, the mRNA expression of the antiapoptotic gene *Bcl-2* was markedly elevated in the 0.4%Trp treatment relative to the C group (*p* < 0.05, Figure 4k).

### 3.6. Intestinal Biological Barrier

The intestinal microbial community structure was characterized via 16S rRNA sequencing, with the corresponding results displayed in Figure 5. The 0.4% and 0.8%Trp groups exhibited a greater count of OTUs relative to the C group (Figure 5a). Consistently, the Simpson and Shannon diversity indices were significantly higher in 0.4% and 0.8%Trp treatments relative to the C group (*p* < 0.05, Figure 5b). Principal coordinate analysis (PCoA) further demonstrated a distinct divergence in gut microbial communities between the 0.4%Trp treatment and the C group (Figure 5c).

**Figure 5:**
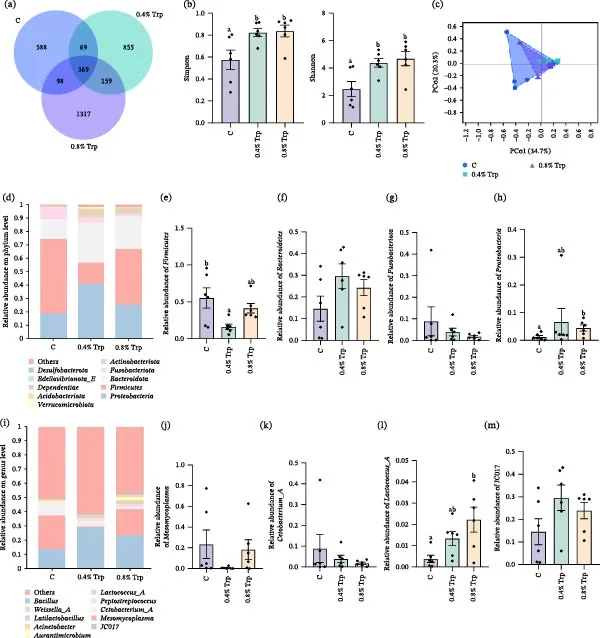
Analysis of gut‐microbiome diversity in largemouth bass. (a) OTU quantity, (b) α‐diversity analysis, and (c) β‐diversity analysis. Intestinal microbiota composition at phylum (d) and genus level (i). Relative abundance of Firmicutes (e), Bacteroidetes (f), Fusobacteria (g), Proteobacteria (h), *Mesomycoplasma* (j), *Cetobacterium_A* (k), *Lactococcus* (l), and *JC017* (m). Data are presented as mean ± SEM (*n* = 6). Values within the same row bearing dissimilar superscript letters differ significantly at *p*  < 0.05.

Proteobacteria, Firmicutes, Bacteroidetes, and Fusobacteria constituted the predominant phyla at the phylum level (Figure 5d). No significant difference was detected in the relative abundance of Bacteroidetes (*p* > 0.05, Figure 5f). The abundance of Fusobacteria declined numerically in the 0.4% and 0.8%Trp treatments compared with group C without significant differences (*p* > 0.05, Figure 5g). The relative abundance of Firmicutes was markedly lower in 0.4%Trp treatment than in the C group (*p* < 0.05, Figure 5e), whereas the 0.8%Trp group exhibited markedly higher Proteobacteria abundance relative to group C (*p* < 0.05, Figure 5h).

At the genus level, the predominant genera comprised *JC017*, *Mesomycoplasma*, *Cetobacterium_A*, and *Lactococcus* (Figure 5i). Fish fed the 0.8%Trp diet revealed a markedly higher relative abundance of *Lactococcus* relative to the C group (*p* < 0.05, Figure 5l). No marked differences in the abundances of *Mesomycoplasma*, *Cetobacterium_A*, and *JC017* were observed across all dietary treatments (*p* > 0.05, Figure 5j,k,m).

### 3.7. Tryptophan Metabolism‐Related Enzymes

As presented in Figure 6, the activities of key enzymes involved in the intestinal tryptophan metabolism of largemouth bass were determined. The IDO1 activity was markedly higher in the 0.4%Trp treatment relative to the C and 0.1%Trp treatment (*p* < 0.05, Figure 6a). Furthermore, all Trp‐supplemented treatments exhibited markedly elevated KYNU and QUIN activities relative to the C group (*p* < 0.05, Figure 6b,c).

**Figure 6:**
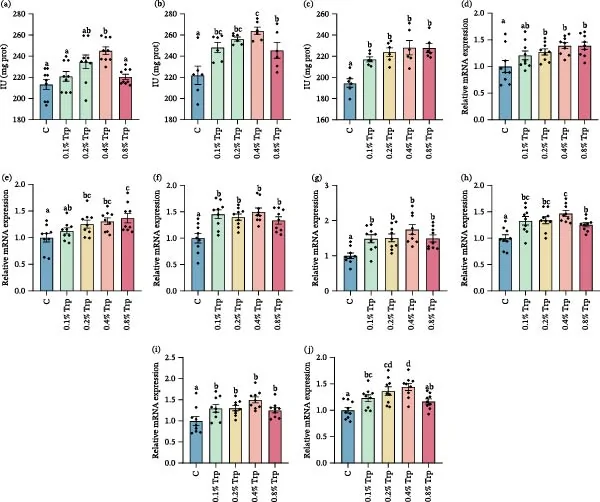
Effects of L‐tryptophan on intestinal tryptophan metabolism‐related enzyme activities (a, b, and c) and gene expression (d, e, f, g, h, i, and j) in largemouth bass. (a) IDO1, (b) KYNU, (c) QUIN, (d) *IDO1*, (e) *KYNU*, (f) *NMNAT1*, (g) *QPRT*, (h) *mTOR*, (i) *S6K1*, and (j) *4EBP1*. Data are presented as mean ± SEM (*n* = 9). Values within the same row bearing dissimilar superscript letters differ significantly at *p*  < 0.05.

Dietary Trp inclusion at 0.2%, 0.4%, and 0.8% significantly elevated the transcript levels of *IDO1* and *KYNU* relative to the C group (*p* < 0.05, Figure 6d,e). Similarly, the mRNA expression level of *4EBP1* was detected in the 0.1%, 0.2%, and 0.4%Trp treatments relative to the C group (*p* < 0.05, Figure 6j). Moreover, the gene expressions of *NMNAT1*, *QPRT*, *mTOR*, and *S6K1* were markedly higher in all Trp‐supplemented treatments relative to the C group (*p* < 0.05, Figure 6f–i).

### 3.8. *A. hydrophila* Challenge Trial

Survival curves of largemouth bass following the *A. hydrophila* challenge are shown in Figure 7. The survival rate reached 100% in the NC group, whereas the C group exhibited a survival rate of 53.33%. The survival values of Trp‐supplemented groups were 66.67% (0.1%Trp), 73.33% (0.2%Trp), 80% (0.4%Trp), and 60% (0.8%Trp), respectively. Collectively, these results indicated that dietary supplementation with 0.4%Trp markedly enhanced the disease resistance of largemouth bass against *A. hydrophila* infection and consequently elevated postchallenge survival.

**Figure 7:**
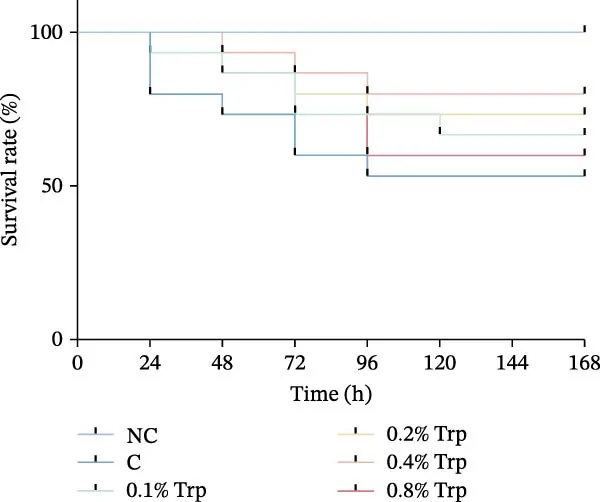
Effects of L‐tryptophan supplementation on survival rate of largemouth bass infected with *Aeromonas hydrophila*.

The serum nonspecific immune parameters of largemouth bass after *A. hydrophila* challenge are listed in Table 7. The LZM activity was markedly higher in the 0.2% and 0.4%Trp treatments than in group C (*p* < 0.05). Consistently, dietary addition with 0.4%Trp markedly increased AKP activity relative to the C group (*p* < 0.05). Besides, ACP activity was significantly increased in all Trp‐supplementation treatments relative to the C group, and the 0.4%Trp treatment reached the highest level (*p* < 0.05).

**Table 7:** Effects of L‐tryptophan supplementation on serum and intestinal antioxidant capacity in largemouth bass challenged with *A. hydrophila*.

Index	Group					
	NC	C	0.1%Trp	0.2%Trp	0.4%Trp	0.8%Trp
*Serum*						
LZM (U/mL)	566.15 ± 11.11^c^	423.56 ± 5.30^a^	451.48 ± 9.66^ab^	456.14 ± 9.64^b^	473.10 ± 8.05^b^	453.92 ± 14.95^ab^
ACP (U/mL)	69.44 ± 0.85^b^	63.68 ± 0.76^a^	71.64 ± 1.06^b^	72.15 ± 0.45^b^	76.02 ± 1.62^c^	70.53 ± 0.87^b^
AKP (U/mL)	62.69 ± 0.85^c^	56.63 ± 0.82^a^	58.27 ± 0.55^ab^	58.62 ± 0.69^ab^	59.64 ± 0.83^b^	57.22 ± 0.49^a^
*Intestinal*						
MDA (nmol/mg prot)	4.11 ± 0.37^a^	5.81 ± 0.29^c^	5.30 ± 0.36^bc^	5.06 ± 0.26^bc^	4.83 ± 0.21^ab^	5.31 ± 0.16^bc^
T‐SOD (U/mg prot)	33.38 ± 1.18^c^	23.51 ± 1.81^a^	27.69 ± 1.26^ab^	30.10 ± 1.40^bc^	31.66 ± 1.89^bc^	28.22 ± 1.07^b^

*Note*: Data are presented as mean ± SEM (*n* = 9). Values within the same row bearing dissimilar superscript letters differ significantly at *p*  < 0.05.

The serum MDA concentration was markedly reduced in the 0.4%Trp treatment relative to group C, while the T‑SOD activities were markedly elevated in the 0.2%, 0.4%, and 0.8%Trp treatments relative to the C group (*p* < 0.05, Table 7). Furthermore, the expression level of *Nrf2* was markedly upregulated in 0.4%Trp treatment versus group C (*p* < 0.05, Figure 8a), and the expression level of *Keap1* was significantly downregulated across all Trp‐supplemented treatments (*p* < 0.05, Figure 8b). Similarly, the expression of *Hsp70* was significantly reduced in the 0.2%, 0.4%, and 0.8%Trp treatments relative to group C (*p* < 0.05, Figure 8c).

**Figure 8:**
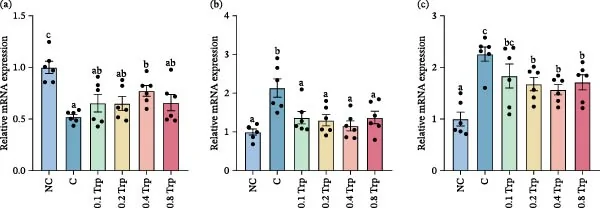
Effects of L‐tryptophan supplementation on intestinal antioxidant gene expression in largemouth bass challenged with *A. hydrophila*. Data are presented as mean ± SEM (*n* = 9). Values within the same row bearing dissimilar superscript letters differ significantly at *p*  < 0.05. (a) *Nrf2*, (b) *Keap1*, and (c) *Hsp70*.

The histopathological observations of intestinal tissues in largemouth bass infected with *A. hydrophila* are revealed in Figure 9. Intestinal villi in the NC group were neatly arranged with intact mucosal, muscular, and serosal layers, and no pathological lesions were observed, whereas obvious destruction of intestinal architecture occurred in group C (Figure 9a). Relative to the C group, the lengths of intestinal villi in the 0.1%, 0.2%, 0.4%, and 0.8%Trp treatments were markedly higher, with the 0.4%Trp treatment showing the most pronounced effect (*p* < 0.05, Figure 9b). Moreover, the muscular layer thickness was markedly higher in the 0.1% and 0.4%Trp groups than that in the C and 0.2%Trp treatments (*p* < 0.05, Figure 9c).

**Figure 9:**
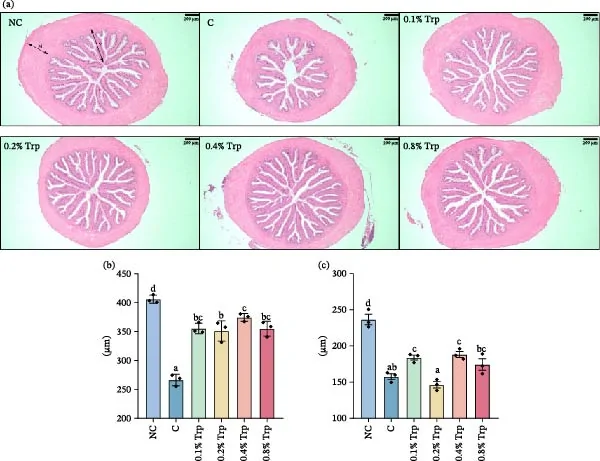
Effects of dietary L‐tryptophan supplementation on intestinal tissue morphology in largemouth bass challenged with *A. hydrophila*. (a) Midgut H&E staining ×50 (*n* = 3), Bar = 200 μm. V: Villus, M: Muscularis. (b) villus height and (c) muscular thickness. Data are presented as mean ± SEM (*n* = 9). Values within the same row bearing dissimilar superscript letters differ significantly at *p*  < 0.05.

Dietary Trp supplementation markedly suppressed the transcript levels of *TNF-α* and *IL-1β* relative to the C group, with the most prominent reduction detected in the 0.4% Trp treatment (*p* < 0.05, Figure 10a,b). Likewise, the expression of *IL-8* was markedly lower in the 0.2% and 0.4%Trp treatments relative to the C group (*p* < 0.05, Figure 10c). By contrast, the expression of antiinflammatory *IL-10* was significantly upregulated in all Trp‐supplemented treatments, peaking in the 0.4%Trp group (*p* < 0.05, Figure 10e). Consistently, the expression level of *TGF‑β1* in the 0.4%Trp treatment was notably increased compared to that in group C (*p* < 0.05, Figure 10d). No significant differences in *IL-22* expression were determined between group C and all four Trp‐supplemental groups (*p* > 0.05, Figure 10f).

**Figure 10:**
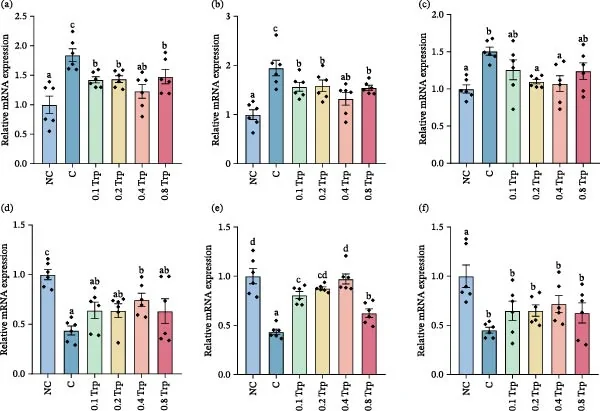
Effects of L‐tryptophan supplementation on the expression of intestinal immune‐related genes in largemouth bass challenged with *A. hydrophila*. (a) *TNF-α*, (b) *IL-1β*, (c) *IL-8*, (d) *TGF-β1*, (e) *IL-10*, and (f) *IL-22*. Data are presented as mean ± SEM (*n* = 9). Values within the same row bearing dissimilar superscript letters differ significantly at *p* < 0.05.

## 4. Discussion

As an essential amino acid for fish, Trp exerts a pivotal regulatory role in growth and metabolic processes [34]. Previous research has demonstrated that optimal dietary Trp supplementation promotes fish growth, whereas both deficient and excessive Trp inclusion can conversely impair growth [35]. In this study, the WGR and SGR of largemouth bass exhibited a pattern of initial increase followed by a decrease with rising dietary Trp levels, which is consistent with previous findings in Jian carp (*Cyprinus carpio var. Jian*) and rainbow trout [36, 37]. This dose‐dependent response is likely mediated by the serotonin (5‐HT) metabolic pathway. As a precursor substrate, tryptophan can be converted into the neurotransmitter 5‐HT by tryptophan hydroxylase [38]. Optimal levels of 5‐HT stimulate feed intake, whereas excessive dietary Trp may lead to excessive cerebral 5‐HT synthesis, which in turn induces satiety, reduces feed intake, and consequently impairs growth. A similar pattern has also been reported in studies on catfish [26] and grass carp (*Ctenopharyngodon idella*) [39]. Moreover, our findings demonstrated that 0.4% dietary Trp inclusion significantly elevated whole‐body crude protein content while reducing crude lipid content in largemouth bass. Such alterations in body composition are directly driven by the conversion efficiency of dietary nutrients, including protein and lipid [40, 41]. Similarly, dietary Trp inclusion has been documented to markedly modulate whole‐body crude protein and lipid levels in mrigal carp (*Cirrhinus mrigala*) [42] and hybrid grouper [20], with consequent effects on growth performance. We postulate that this regulatory effect may be attributed to the Trp‐mediated activation of the *mTOR/S6K1/4EBP1* signaling pathway, which governs protein synthesis. As a pivotal regulator of protein anabolism, *mTOR* mainly triggers downstream signaling via the phosphorylation of *S6K1* and *4EBP1* upon activation, ultimately driving protein synthesis [43]. Notably, such regulatory effects primarily occur at the posttranslational level rather than transcriptional modulation. Consistent with our molecular observations, dietary tyrosine supplementation significantly upregulated intestinal *S6K1* mRNA expression in largemouth bass, accompanied by a parallel increase in *4EBP1* transcription. Supportively, previous studies on grass carp have also demonstrated that dietary Trp supplementation significantly elevated muscle protein content in strong correlation with the upregulation of *S6K1* gene expression [39].

Antioxidant capacity is a core physiological function that maintains systemic redox homeostasis by scavenging excess ROS in a timely manner, thereby alleviating damage to cellular structure and function [22]. In the present trial, the activities of CAT, SOD, and GSH‐Px as well as T‐AOC levels in both serum and intestinal tissues of largemouth bass exhibited an initial increase followed by a decline as dietary Trp levels rose. By contrast, the MDA content showed the opposite pattern, decreasing first and then increasing. These findings are consistent with those documented in prior studies on blunt snout bream (*Megalobrama amblycephala*) [35] and rainbow trout [44], collectively indicating that Trp modulates the antioxidant system in fish. Furthermore, an imbalanced dietary amino acid composition can induce ROS accumulation, which ultimately damages cellular structure, protein function, and tissue integrity [14]. This might partly explain the diminished antioxidant capacity detected in the 0.8% Trp group of the present trial. It is well established that the *Nrf2/Keap1* axis acts as a central regulator of antioxidant defense responses to oxidative stress. In this study, with increasing dietary Trp levels, the mRNA expression of *Nrf2* exhibited a pattern of an initial increase followed by a decrease, whereas *Keap1* showed an opposite trend of an initial decrease followed by an increase. Activation of *Nrf2* signaling is known to attenuate free‐radical‐induced tissue damage, thereby reinforcing the host antioxidant defense system and enhancing overall resistance to oxidative stress [45]. This regulatory mechanism appears to be conserved as a similar modulatory effect of Trp on the *Nrf2/Keap1* axis has been documented in grass carp [46].

Beyond its well‐recognized function in nutrient digestion and absorption, the fish intestine serves as the first line of defense against pathogenic invasion. The structural integrity of the intestinal barrier is directly essential for maintaining normal physiological functions [30, 47]. Accumulating evidence indicates that the fish intestinal barrier consists of physical, chemical, immune, and biological barriers, which collectively maintain intestinal homeostasis [29]. The intestinal physical barrier is formed by the intestinal mucosal layer and epithelial cells and comprises apical tight junctions, desmosomes, adherens junctions, and gap junctions, among other structures [48]. Crucially, the efficiency of intestinal nutrient absorption is largely determined by the morphological structure of intestinal villi and the size of the effective absorptive surface area [49]. In this research, fish fed diets with 0.1%, 0.2%, and 0.4%Trp significantly enhanced both the intestinal villus height and muscular layer thickness relative to the C group. Such patterns are congruent with findings from prior studies conducted on triploid crucian carp (*Carassius auratus gibelio*) [50] and yellow catfish (*Pelteobagrus fulvidraco ♀ × Pelteobagrus vachelli ♂*) [28]. Taken together, these data indicate that dietary Trp supplementation increases intestinal villus height, thereby improving intestinal health and promoting nutrient absorption. Furthermore, tight junctions are intercellular junctions between adjacent epithelial cells, composed of multiple tight junction proteins including ZOs, occludin, and claudins. By modulating intestinal barrier permeability, they facilitate the selective uptake of dietary nutrients while impeding the translocation of detrimental substances into the host’s systemic circulation [51, 52]. In our research, fish fed Trp‐supplemented diets exhibited significantly elevated transcript levels of core intestinal tight junction genes (*ZO-1*, *Claudin-1*, and *Claudin-4*) relative to the C group, with the most robust upregulation detected in the 0.4% Trp treatment. These findings suggest that Trp can ameliorate intestinal physical barrier damage in largemouth bass by regulating the expression of tight junction proteins. Similarly, a study in grass carp [39] indicated that optimal dietary Trp levels significantly upregulated the expression of *Occludin*, *ZO-1*, *Claudin-b*, *Claudin-c*, and *Claudin-3* in the intestine. Additionally, our results revealed that the activities of trypsin, lipase, and α‐amylase in the foregut were markedly higher in the 0.4%Trp treatment than in the C group. Consistently, dietary Trp supplementation has been demonstrated to markedly increase protease activities in the gut and liver of Jian carp [36]. These findings suggest that Trp can promote nutrient digestion and absorption by enhancing digestive enzyme activities [25].

The intestinal immune barrier of fish primarily relies on cytokines secreted by immune cells [53]. As critical signaling mediators of intestinal inflammation, cytokines are generally classified into proinflammatory cytokines (e.g., TNF‐α, IL‐1β, and IL‐8) and antiinflammatory cytokines (e.g., TGF‐β and IL‐10) [54]. These inflammatory factors not only activate immune cells to participate in immune regulation but also stimulate the secretion of other inflammatory factors, thereby initiating or aggravating inflammatory responses [55]. In this study, intestinal transcript levels of *TNF‑α*, *IL‑1β*, and *IL‑8* were markedly downregulated across all Trp‑supplemented groups compared with the C group. This indicates that dietary Trp supplementation effectively repressed intestinal proinflammatory cytokine expression and improved intestinal immune status. Furthermore, TGF‐β exerts a vital function in regulating immune responses. When the body is attacked by viruses, pathogenic bacteria, or immune stimulants, the expression of TGF‐β1 in the body significantly increases. Similarly, as a canonical antiinflammatory cytokine, IL‐10 modulates inflammatory reactions by inhibiting the transcription of proinflammatory mediators, such as TNF‐α and IL‐1β [56]. In this study, largemouth bass in the 0.4%Trp group exhibited significantly higher intestinal transcript levels of antiinflammatory cytokines (*IL‑10* and *TGF‑β1*) than the C group. The present observations are in agreement with earlier findings in yellow catfish [28].

Furthermore, the interaction between tryptophan metabolites and the intestinal aryl hydrocarbon receptor (AHR) has also been widely implicated in the pathogenesis of intestinal inflammation [57]. Among them, CYP1A1 is the classic target gene of AHR, and its function involves immune homeostasis regulation and the response process to pathogenic damage [58]. The findings of the current study reveal that the transcript levels of *AHR* and *CYP1A1* in the intestine of the Trp‐supplemented groups were markedly higher relative to those of the C group. Meanwhile, the intestinal AHR‐IL‐22 signal serves dual roles in defending against pathogen invasion [59] and strengthening the tight junction function to preserve intestinal barrier integrity [60]. Consistently, previous research in mice has shown that Trp supplementation markedly mitigates acute hypoxic stress‐induced intestinal tissue damage and apoptosis by improving barrier integrity and reducing intestinal permeability [61]. Collectively, these findings suggested that dietary Trp supplementation may modulate proliferation and differentiation of the intestinal immune system through the Trp‐AHR signaling pathway, thereby facilitating the preservation of gut homeostasis.

As a crucial digestive and immune organ in fish, the intestine harbors large and diverse microbial communities, whose composition and stability are closely linked to intestinal health in aquatic animals [62]. Prior investigations have documented that dietary nutritional components exert significant impacts on the structure and function of the intestinal microbiota [63]. The observations from this study revealed that dietary Trp supplementation markedly increased the number of operational taxonomic units (OTUs) and alpha diversity indices (Shannon index and Simpson index) in the intestinal microbiota, suggesting that dietary Trp supplementation enhances both the species richness and alpha diversity within the intestinal microbiota [64]. Consistent results were observed in crayfish (*Procambarus clarkii*), in which dietary supplementation with 0.43% Trp significantly elevated both the Shannon and Simpson indices [65]. Previous reports have demonstrated that the intestinal microbiota of largemouth bass is dominated by Proteobacteria, Firmicutes, and Bacteroidetes at the phylum level [66], which coincides with the results obtained herein. Furthermore, our findings indicate that dietary Trp supplementation elevated the relative abundance of Bacteroidetes. Bacteroidetes are of great importance to carbohydrate degradation and protein metabolism, thereby enhancing the host’s efficiency in nutrient utilization. This may partially explain the improved feed utilization observed in largemouth bass after Trp supplementation. Additionally, dietary Trp supplementation significantly enriched the relative abundance of Lactococcus. Accumulating evidence demonstrated that Lactococcus can metabolize Trp into indole‐3‐lactic acid, which activates the cellular AHR and promotes cell differentiation and intestinal barrier repair [67]. This may represent one of the core mechanisms underlying the Trp‐induced enhancement of intestinal barrier function. Taken together, dietary Trp supplementation effectively enhances the gut barrier integrity and improves intestinal health in largemouth bass by strengthening the physical, immune, and biological barriers, thereby contributing to improved growth performance.

Currently, persistent deterioration of aquatic environments driven by high‐density aquaculture has markedly elevated disease prevalence, representing a key constraint restricting the healthy and sustainable advancement of the largemouth bass sector [68]. *A. hydrophila* is one of the major pathogenic bacteria responsible for diseases in freshwater fish, which can induce multiple disorders, including septicemia and enteritis and even cause mortality [69, 70]. As a core immune organ in fish, the intestine serves a pivotal role in defense against pathogen invasion [71]. In this study, Trp‐supplemented diets effectively improved the intestinal health of largemouth bass. Following the *A. hydrophila* challenge, the survival rate of fish in the 0.4%Trp group was 50.02% greater compared with the C group. This indicates that Trp significantly enhances the resistance of largemouth bass to *A. hydrophila* infection and thereby effectively improves postinfection survival. This finding is consistent with previous results reported in Jian carp [36]. Furthermore, accumulating evidence indicates that fish can defend against pathogens via circulating nonspecific immune factors, LZM, AKP, and ACP [72]. Specifically, LZM exerts direct bactericidal activity by disrupting the cell wall of pathogenic bacteria. ACP, a core component of lysosomes, is commonly utilized as a biomarker for macrophage activation, whereas AKP is an extracellular enzyme that hydrolyzes a broad range of organophosphate compounds [73]. In this study, serum activities of LZM, AKP, and ACP were all elevated in Trp‑supplemented groups, suggesting that dietary Trp enhances the resistance of largemouth bass to *A. hydrophila* infection by boosting nonspecific immune competence. Similarly, dietary Trp supplementation has also been reported to increase serum LZM activity and enhance macrophage phagocytic capacity in Nile tilapia (*Oreochromis niloticus*) [74]. In addition, this study also demonstrated that dietary Trp supplementation increased the T‐SOD activity in the intestines after bacterial challenge and reduced the MDA content. It also restored the transcript levels of *Keap1* and *Nrf2* to normal. These results indicate that Trp may strengthen systemic antioxidant capacity and alleviate redox imbalance by modulating the Keap1–Nrf2 signal pathway and downregulating *Hsp70* transcription. Additionally, to elucidate the regulatory impacts of Trp on intestinal inflammatory responses in largemouth bass following *A. hydrophila* challenge, we systematically evaluated the expression changes of intestinal proinflammatory factors (*TNF-α*, *IL-8*, and *IL-1β*) and antiinflammatory factors (*TGF-β1*, *IL-10*, and *IL-22*). These results showed that 0.4% Trp‐supplemented diets markedly reduced the mRNA expression of gut proinflammatory factors induced by *A. hydrophila* infection and elevated the expression of antiinflammatory cytokine genes. These findings indicate that Trp enhances resistance to *A. hydrophila* infection by modulating the expression of immune cytokine‐related genes. Consistently, a previous study in yellow catfish demonstrated that Trp supplementation in low‐fishmeal diets downregulated intestinal proinflammatory cytokine expression and elevated antiinflammatory cytokine levels [28]. Collectively, the above results indicate that a 0.4% Trp‐supplemented diet can elevate the activities of serum nonspecific immune enzymes and intestinal antioxidant enzymes in largemouth bass following infection with *A. hydrophila*, thereby mitigating oxidative damage. In addition, Trp supplementation ameliorates intestinal structural injury induced by infection and concurrently downregulates the expression of proinflammatory genes while upregulating that of antiinflammatory genes, ultimately strengthening systemic immune function and enhancing the antiinfective capacity of largemouth bass.

## 5. Conclusion

In conclusion, quadratic regression analysis estimated the optimal dietary Trp inclusion level for maximizing the growth performance of largemouth bass to be 0.49%. Supplementation with 0.4%Trp effectively activated the intestinal Nrf2/Keap1 signaling pathway, enhanced the antioxidant status, and alleviated oxidative damage under both normal culture and *A. hydrophila* infection. Furthermore, Trp supplementation improved the intestinal morphological integrity, upregulated the expression of tight junction genes, modulated the inflammatory responses, and optimized the intestinal microbial composition. Following an *A. hydrophila* challenge, dietary Trp not only significantly increased the fish survival rate but also enhanced serum nonspecific immune capacity. Collectively, these findings demonstrate that 0.4% dietary Trp supplementation achieves the optimal effects on fish growth, intestinal homeostasis, and disease resistance. These findings provide a theoretical basis for the application of Trp as a functional feed additive for healthy largemouth bass farming.

## Author Contributions

**Xindang Zhang and Hengzhi Wang:** formal analysis, writing – original draft, investigation, writing – review and editing. **Chongchong Guo and Shikun Feng:** data curation, validation, writing – original draft. **Xue Tian, Yanmin Zhang, and Xulu Chang:** formal analysis, resources, data curation. **Guokun Yang, Mohammed A. E. Naiel, and Xiaolin Meng:** conceptualization, supervision, writing – review and editing.

## Funding

This research was financially supported by the National Key R&D Program of China (Grant 2024YFD2402005), the National Natural Science Foundation of China (Grants 32503210, 32273149, and 32303020), the Henan Provincial Natural Science Foundation (Grant 252300423625), and the Key Research Project of Henan Provincial Universities (Grant 26B240002).

## Disclosure

All authors contributed to the article and approved the submitted version.

## Ethics Statement

All animal protocols were approved by the Animal Administration and Ethics Committee of the Academic Committee of Henan Normal University (Number HNSD‐2025‐08‐18).

## Conflicts of Interest

The authors declare no conflicts of interest.

## Data Availability

The data will be made available upon request.
